# 2-(1,5-Diphenyl-1*H*-pyrazol-3-yl­oxy)-1-(2-sulfanyl­idene-1,3-thia­zolidin-3-yl)­ethanone

**DOI:** 10.1107/S1600536812029959

**Published:** 2012-07-10

**Authors:** Yi Li, Yuan-Yuan Liu, Xiao-Hui Xiong, Ping Wei

**Affiliations:** a College of Biotechnology and Pharmaceutical Engineering, Nanjing University of Technology, Nanjing 210009, People’s Republic of China; b Department of Chemical and Pharmaceutical Engineering, Southeast University ChengXian College, Nanjing 210088, People’s Republic of China; c College of Food Science and Light Industry, Nanjing University of Technology, Nanjing 210009, People’s Republic of China

## Abstract

The title compound, C_20_H_17_N_3_O_2_S_2_, was synthesized by the reaction of 2-(1,5-diphenyl-1*H*-pyrazol-3-yl­oxy)acetic acid and thia­zolidine-2-thione. The C-linked benzene ring, N-linked benzene ring and thia­zolidine-2-thione ring are twisted 31.33 (2), 62.87 (1) and 82.71 (2)°, respectively, from the plane of the bridging 1*H*-pyrazole ring. The phenyl rings are oriented at a dihedral angle of 72.16 (2)°.

## Related literature

For pyrazol derivative bioactivities, see: Aly (2009 ▶); Meegalla *et al.* (2004 ▶); Morimoto *et al.* (1990 ▶). For a related structure, see: Goodman *et al.* (1971 ▶). For bond lengths, see: Allen *et al.* (1987 ▶). For the literature method used for preparation, see: Liu *et al.* (2011 ▶).
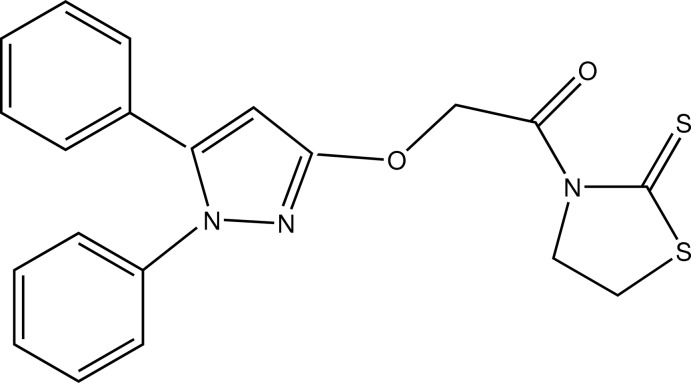


## Experimental

### 

#### Crystal data

C_20_H_17_N_3_O_2_S_2_
*M* *_r_* = 395.49
Monoclinic, 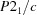

*a* = 12.813 (3) Å
*b* = 16.453 (3) Å
*c* = 8.9470 (18) Å
β = 97.68 (3)°
*V* = 1869.2 (7) Å^3^
*Z* = 4
Mo *K*α radiation
μ = 0.31 mm^−1^
*T* = 293 K
0.30 × 0.20 × 0.10 mm

#### Data collection

Enraf–Nonius CAD-4 diffractometer
Absorption correction: ψ scan (North *et al.*, 1968 ▶) *T*_min_ = 0.914, *T*_max_ = 0.970
3625 measured reflections
3391 independent reflections
2202 reflections with *I* > 2σ(*I*)
*R*_int_ = 0.024
3 standard reflections every 200 reflections intensity decay: 1%

#### Refinement

*R*[*F* ^2^ > 2σ(*F* ^2^)] = 0.068
*wR*(*F* ^2^) = 0.149
*S* = 1.08
3391 reflections
244 parameters
2 restraints
H-atom parameters constrained
Δρ_max_ = 0.65 e Å^−3^
Δρ_min_ = −0.68 e Å^−3^

### 

Data collection: *CAD-4 Software* (Enraf–Nonius, 1985 ▶); cell refinement: *CAD-4 Software*; data reduction: *XCAD4* (Harms & Wocadlo, 1995 ▶); program(s) used to solve structure: *SHELXS97* (Sheldrick, 2008 ▶); program(s) used to refine structure: *SHELXL97* (Sheldrick, 2008 ▶); molecular graphics: *SHELXTL* (Sheldrick, 2008 ▶); software used to prepare material for publication: *SHELXTL*.

## Supplementary Material

Crystal structure: contains datablock(s) I, global. DOI: 10.1107/S1600536812029959/bq2369sup1.cif

Structure factors: contains datablock(s) I. DOI: 10.1107/S1600536812029959/bq2369Isup2.hkl

Supplementary material file. DOI: 10.1107/S1600536812029959/bq2369Isup3.cml

Additional supplementary materials:  crystallographic information; 3D view; checkCIF report

## Figures and Tables

**Fig. 1.**
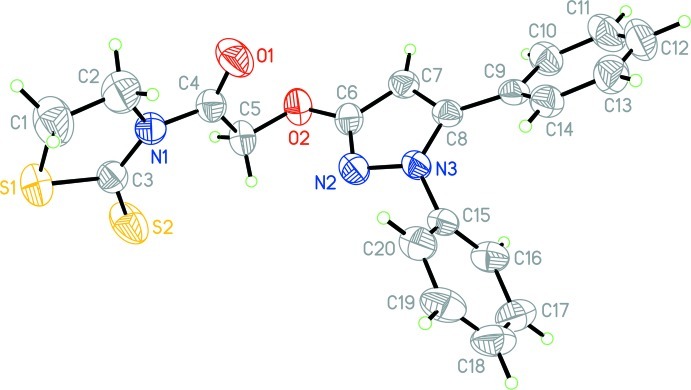
The molecular structure of (I), with the atom-numbering scheme. Displacement ellipsoids are drawn at the 50% probability level.

## supplementary crystallographic information

### Comment

Since the discovery of the strobilurin fungicide pyraclostrobin by BASF scientists, 1*H*-pyrazol-3-oxy derivatives have attracted considerable attention in chemical and medicinal research due to their low mammalian toxicity and diverse bioactivities such as fungicidal (Aly, 2009), insecticidal (Meegalla *et al.*, 2004) and herbicidal (Morimoto *et al.*, 1990) activities. However, very few representatives of biologically active 2-(1,5-diaryl-1*H*-pyrazol-3-yloxy)-1-(2-thioxothiazolidin-3-yl)ethanone derivatives have hitherto been described in the literature. We report here the crystal structure of the title compound, (I).

In the molecule of (I), (Fig.1), the bond lengths (Allen *et al.*, 1987) and angles are within normal ranges. The bond length of N1—C4 (1.409 (5) Å) is longer than normal N—C amide bond (1.325–1.352 Å) (Goodman *et al.*, 1971). The C-linked benzene ring A (C9—C14), N-linked benzene ring B (C15—C20), and thiazolidine-2-thione ring (N1/S1/C1—C3) are twisted 31.33 °, 62.87 °, and 82.71 ° from the plane of the bridge 1*H*-pyrazol ring (N2/N3/C6—C8), respectively. Rings A and B are, of course, planar and the dihedral angle between them is 72.16 °.

### Experimental

The title compound, (I) was prepared by the literature method (Liu *et al.*, 2011). Crystals suitable for X-ray analysis were obtained by dissolving (I) (0.5 g) in ethyl acetate (20 ml) and evaporating the solvent slowly at room temperature for about 7 d.

### Refinement

H atoms were positioned geometrically, with C—H = 0.93 and 0.97 Å for aromatic and methylene H, respectively, and constrained to ride on their parent atoms, with *U*_iso_(H) = *xU*_eq_(C), where *x* = 1.2 for aromatic H, and *x* = 1.5 for other H.

### Crystal data

**Table:**

C_20_H_17_N_3_O_2_S_2_	*F*(000) = 824
*M**_r_* = 395.49	*D*_x_ = 1.405 Mg m^−^^3^
Monoclinic, *P*2_1_/*c*	Mo *K*α radiation, λ = 0.71073 Å
Hall symbol: -P 2ybc	Cell parameters from 25 reflections
*a* = 12.813 (3) Å	θ = 9–13°
*b* = 16.453 (3) Å	µ = 0.31 mm^−^^1^
*c* = 8.9470 (18) Å	*T* = 293 K
β = 97.68 (3)°	Needle, yellow
*V* = 1869.2 (7) Å^3^	0.30 × 0.20 × 0.10 mm
*Z* = 4	

### Data collection

**Table:**

Enraf–Nonius CAD-4 diffractometer	2202 reflections with *I* > 2σ(*I*)
Radiation source: fine-focus sealed tube	*R*_int_ = 0.024
Graphite monochromator	θ_max_ = 25.3°, θ_min_ = 1.6°
ω/2θ scans	*h* = −15→15
Absorption correction: ψ scan (North *et al.*, 1968)	*k* = −19→0
*T*_min_ = 0.914, *T*_max_ = 0.970	*l* = 0→10
3625 measured reflections	3 standard reflections every 200 reflections
3391 independent reflections	intensity decay: 1%

### Refinement

**Table:**

Refinement on *F*^2^	Primary atom site location: structure-invariant direct methods
Least-squares matrix: full	Secondary atom site location: difference Fourier map
*R*[*F*^2^ > 2σ(*F*^2^)] = 0.068	Hydrogen site location: inferred from neighbouring sites
*wR*(*F*^2^) = 0.149	H-atom parameters constrained
*S* = 1.08	*w* = 1/[σ^2^(*F*_o_^2^) + (0.0324*P*)^2^ + 2.7384*P*] where *P* = (*F*_o_^2^ + 2*F*_c_^2^)/3
3391 reflections	(Δ/σ)_max_ < 0.001
244 parameters	Δρ_max_ = 0.65 e Å^−^^3^
2 restraints	Δρ_min_ = −0.68 e Å^−^^3^

### Special details

**Table:**

Geometry. All e.s.d.'s (except the e.s.d. in the dihedral angle between two l.s. planes) are estimated using the full covariance matrix. The cell e.s.d.'s are taken into account individually in the estimation of e.s.d.'s in distances, angles and torsion angles; correlations between e.s.d.'s in cell parameters are only used when they are defined by crystal symmetry. An approximate (isotropic) treatment of cell e.s.d.'s is used for estimating e.s.d.'s involving l.s. planes.
Refinement. Refinement of *F*^2^ against ALL reflections. The weighted *R*-factor *wR* and goodness of fit *S* are based on *F*^2^, conventional *R*-factors *R* are based on *F*, with *F* set to zero for negative *F*^2^. The threshold expression of *F*^2^ > σ(*F*^2^) is used only for calculating *R*-factors(gt) *etc*. and is not relevant to the choice of reflections for refinement. *R*-factors based on *F*^2^ are statistically about twice as large as those based on *F*, and *R*- factors based on ALL data will be even larger.

### Fractional atomic coordinates and isotropic or equivalent isotropic displacement parameters (Å^2^)

**Table:**

	*x*	*y*	*z*	*U*_iso_*/*U*_eq_	
S1	0.53480 (10)	0.88849 (7)	0.53157 (15)	0.0611 (4)	
S2	0.62593 (11)	0.72831 (9)	0.50196 (19)	0.0775 (5)	
O1	0.3356 (3)	0.6778 (2)	0.7213 (4)	0.0675 (10)	
O2	0.4300 (2)	0.53904 (17)	0.6832 (3)	0.0519 (8)	
N1	0.4433 (3)	0.7614 (2)	0.6153 (4)	0.0431 (8)	
N2	0.3178 (3)	0.5311 (2)	0.4521 (4)	0.0476 (9)	
N3	0.2289 (3)	0.4864 (2)	0.4065 (4)	0.0434 (8)	
C1	0.4111 (4)	0.9057 (3)	0.5959 (6)	0.0849 (18)	
H1A	0.4193	0.9439	0.6792	0.102*	
H1B	0.3617	0.9284	0.5152	0.102*	
C2	0.3715 (4)	0.8282 (3)	0.6449 (5)	0.0704 (15)	
H2B	0.3019	0.8180	0.5913	0.084*	
H2C	0.3660	0.8305	0.7519	0.084*	
C3	0.5321 (3)	0.7847 (3)	0.5549 (4)	0.0445 (10)	
C4	0.4130 (3)	0.6833 (3)	0.6580 (5)	0.0462 (11)	
C5	0.4775 (3)	0.6096 (2)	0.6282 (5)	0.0478 (11)	
H5A	0.4802	0.6042	0.5208	0.057*	
H5B	0.5489	0.6155	0.6787	0.057*	
C6	0.3442 (3)	0.5100 (2)	0.5956 (5)	0.0423 (10)	
C7	0.2761 (3)	0.4523 (2)	0.6429 (5)	0.0426 (10)	
H7A	0.2798	0.4285	0.7378	0.051*	
C8	0.2024 (3)	0.4379 (2)	0.5198 (4)	0.0384 (9)	
C9	0.1076 (3)	0.3868 (2)	0.5110 (4)	0.0405 (10)	
C10	0.1077 (4)	0.3190 (3)	0.6039 (5)	0.0530 (12)	
H10A	0.1695	0.3037	0.6639	0.064*	
C11	0.0169 (4)	0.2743 (3)	0.6081 (6)	0.0685 (15)	
H11A	0.0176	0.2294	0.6713	0.082*	
C12	−0.0750 (4)	0.2962 (3)	0.5185 (7)	0.0693 (15)	
H12A	−0.1360	0.2660	0.5213	0.083*	
C13	−0.0762 (4)	0.3624 (3)	0.4254 (6)	0.0597 (13)	
H13A	−0.1381	0.3770	0.3648	0.072*	
C14	0.0137 (3)	0.4070 (3)	0.4214 (5)	0.0492 (11)	
H14A	0.0120	0.4518	0.3576	0.059*	
C15	0.1921 (3)	0.4838 (3)	0.2477 (4)	0.0418 (10)	
C16	0.1999 (4)	0.4125 (3)	0.1711 (5)	0.0528 (12)	
H16A	0.2262	0.3659	0.2217	0.063*	
C17	0.1679 (4)	0.4109 (3)	0.0157 (5)	0.0610 (13)	
H17A	0.1717	0.3629	−0.0382	0.073*	
C18	0.1307 (4)	0.4805 (4)	−0.0572 (5)	0.0628 (14)	
H18A	0.1095	0.4795	−0.1608	0.075*	
C19	0.1246 (4)	0.5515 (3)	0.0212 (5)	0.0593 (13)	
H19A	0.1000	0.5985	−0.0295	0.071*	
C20	0.1549 (3)	0.5535 (3)	0.1751 (5)	0.0519 (11)	
H20A	0.1502	0.6015	0.2288	0.062*	

### Atomic displacement parameters (Å^2^)

**Table:**

	*U*^11^	*U*^22^	*U*^33^	*U*^12^	*U*^13^	*U*^23^
S1	0.0605 (8)	0.0463 (7)	0.0763 (9)	−0.0077 (6)	0.0087 (6)	0.0054 (7)
S2	0.0616 (8)	0.0618 (9)	0.1185 (13)	0.0057 (7)	0.0472 (8)	0.0103 (9)
O1	0.055 (2)	0.063 (2)	0.090 (3)	−0.0044 (17)	0.0316 (19)	0.0053 (19)
O2	0.0529 (18)	0.0451 (17)	0.0544 (19)	−0.0096 (14)	−0.0054 (15)	0.0072 (15)
N1	0.0407 (19)	0.042 (2)	0.048 (2)	0.0003 (16)	0.0080 (16)	−0.0015 (17)
N2	0.048 (2)	0.050 (2)	0.044 (2)	−0.0088 (18)	0.0075 (17)	0.0045 (18)
N3	0.046 (2)	0.048 (2)	0.0357 (19)	−0.0114 (17)	0.0056 (16)	0.0052 (16)
C1	0.077 (4)	0.053 (3)	0.130 (5)	0.006 (3)	0.037 (4)	0.001 (3)
C2	0.067 (3)	0.050 (3)	0.099 (4)	0.009 (3)	0.031 (3)	−0.008 (3)
C3	0.043 (2)	0.048 (3)	0.041 (2)	−0.003 (2)	0.0004 (19)	0.001 (2)
C4	0.041 (2)	0.049 (3)	0.048 (3)	−0.006 (2)	0.004 (2)	−0.001 (2)
C5	0.043 (2)	0.040 (2)	0.060 (3)	−0.007 (2)	0.004 (2)	0.000 (2)
C6	0.044 (2)	0.034 (2)	0.049 (3)	0.0006 (19)	0.005 (2)	0.000 (2)
C7	0.050 (3)	0.042 (2)	0.037 (2)	−0.001 (2)	0.007 (2)	0.0033 (19)
C8	0.042 (2)	0.037 (2)	0.039 (2)	0.0023 (18)	0.0122 (19)	0.0011 (18)
C9	0.045 (2)	0.041 (2)	0.038 (2)	−0.0004 (19)	0.0165 (19)	−0.005 (2)
C10	0.055 (3)	0.047 (3)	0.059 (3)	−0.001 (2)	0.015 (2)	0.006 (2)
C11	0.078 (4)	0.050 (3)	0.084 (4)	−0.009 (3)	0.034 (3)	0.012 (3)
C12	0.055 (3)	0.061 (3)	0.096 (4)	−0.017 (3)	0.027 (3)	−0.010 (3)
C13	0.051 (3)	0.062 (3)	0.068 (3)	−0.004 (2)	0.013 (2)	−0.012 (3)
C14	0.050 (3)	0.048 (3)	0.052 (3)	−0.002 (2)	0.017 (2)	−0.004 (2)
C15	0.043 (2)	0.050 (3)	0.033 (2)	−0.006 (2)	0.0097 (18)	0.003 (2)
C16	0.061 (3)	0.056 (3)	0.043 (3)	0.005 (2)	0.012 (2)	0.007 (2)
C17	0.064 (3)	0.075 (4)	0.044 (3)	0.004 (3)	0.010 (2)	−0.010 (3)
C18	0.053 (3)	0.099 (4)	0.037 (3)	0.005 (3)	0.006 (2)	0.008 (3)
C19	0.058 (3)	0.072 (3)	0.049 (3)	0.009 (3)	0.011 (2)	0.022 (3)
C20	0.056 (3)	0.052 (3)	0.050 (3)	0.002 (2)	0.015 (2)	0.004 (2)

### Geometric parameters (Å, º)

**Table:**

S1—C3	1.721 (4)	C8—C9	1.470 (5)
S1—C1	1.779 (5)	C9—C10	1.391 (6)
S2—C3	1.639 (4)	C9—C14	1.395 (6)
O1—C4	1.209 (5)	C10—C11	1.382 (6)
O2—C6	1.349 (5)	C10—H10A	0.9300
O2—C5	1.428 (5)	C11—C12	1.380 (7)
N1—C3	1.378 (5)	C11—H11A	0.9300
N1—C4	1.409 (5)	C12—C13	1.369 (7)
N1—C2	1.479 (5)	C12—H12A	0.9300
N2—C6	1.330 (5)	C13—C14	1.371 (6)
N2—N3	1.371 (4)	C13—H13A	0.9300
N3—C8	1.369 (5)	C14—H14A	0.9300
N3—C15	1.437 (5)	C15—C16	1.369 (6)
C1—C2	1.461 (7)	C15—C20	1.372 (6)
C1—H1A	0.9700	C16—C17	1.397 (6)
C1—H1B	0.9700	C16—H16A	0.9300
C2—H2B	0.9700	C17—C18	1.371 (7)
C2—H2C	0.9700	C17—H17A	0.9300
C4—C5	1.513 (6)	C18—C19	1.371 (7)
C5—H5A	0.9700	C18—H18A	0.9300
C5—H5B	0.9700	C19—C20	1.380 (6)
C6—C7	1.393 (5)	C19—H19A	0.9300
C7—C8	1.372 (6)	C20—H20A	0.9300
C7—H7A	0.9300		
			
C3—S1—C1	94.9 (2)	N3—C8—C7	106.4 (3)
C6—O2—C5	116.1 (3)	N3—C8—C9	125.4 (4)
C3—N1—C4	129.3 (4)	C7—C8—C9	128.1 (4)
C3—N1—C2	115.3 (4)	C10—C9—C14	117.8 (4)
C4—N1—C2	115.4 (3)	C10—C9—C8	119.4 (4)
C6—N2—N3	103.9 (3)	C14—C9—C8	122.5 (4)
C8—N3—N2	111.8 (3)	C11—C10—C9	120.6 (5)
C8—N3—C15	129.3 (3)	C11—C10—H10A	119.7
N2—N3—C15	117.4 (3)	C9—C10—H10A	119.7
C2—C1—S1	108.6 (4)	C12—C11—C10	120.1 (5)
C2—C1—H1A	110.0	C12—C11—H11A	120.0
S1—C1—H1A	110.0	C10—C11—H11A	120.0
C2—C1—H1B	110.0	C13—C12—C11	120.0 (5)
S1—C1—H1B	110.0	C13—C12—H12A	120.0
H1A—C1—H1B	108.4	C11—C12—H12A	120.0
C1—C2—N1	110.2 (4)	C12—C13—C14	120.0 (5)
C1—C2—H2B	109.6	C12—C13—H13A	120.0
N1—C2—H2B	109.6	C14—C13—H13A	120.0
C1—C2—H2C	109.6	C13—C14—C9	121.4 (4)
N1—C2—H2C	109.6	C13—C14—H14A	119.3
H2B—C2—H2C	108.1	C9—C14—H14A	119.3
N1—C3—S2	129.2 (3)	C16—C15—C20	121.6 (4)
N1—C3—S1	110.9 (3)	C16—C15—N3	118.9 (4)
S2—C3—S1	120.0 (3)	C20—C15—N3	119.4 (4)
O1—C4—N1	117.9 (4)	C15—C16—C17	118.8 (4)
O1—C4—C5	121.6 (4)	C15—C16—H16A	120.6
N1—C4—C5	120.4 (4)	C17—C16—H16A	120.6
O2—C5—C4	108.8 (3)	C18—C17—C16	119.7 (5)
O2—C5—H5A	109.9	C18—C17—H17A	120.1
C4—C5—H5A	109.9	C16—C17—H17A	120.1
O2—C5—H5B	109.9	C19—C18—C17	120.5 (4)
C4—C5—H5B	109.9	C19—C18—H18A	119.7
H5A—C5—H5B	108.3	C17—C18—H18A	119.7
N2—C6—O2	123.4 (4)	C18—C19—C20	120.3 (5)
N2—C6—C7	112.5 (4)	C18—C19—H19A	119.9
O2—C6—C7	124.1 (4)	C20—C19—H19A	119.9
C8—C7—C6	105.4 (4)	C15—C20—C19	119.1 (5)
C8—C7—H7A	127.3	C15—C20—H20A	120.5
C6—C7—H7A	127.3	C19—C20—H20A	120.5
			
C6—N2—N3—C8	−0.9 (4)	C15—N3—C8—C9	−18.9 (6)
C6—N2—N3—C15	−168.3 (4)	C6—C7—C8—N3	0.0 (4)
C3—S1—C1—C2	3.6 (2)	C6—C7—C8—C9	−174.8 (4)
S1—C1—C2—N1	−3.8 (3)	N3—C8—C9—C10	155.4 (4)
C3—N1—C2—C1	2.3 (4)	C7—C8—C9—C10	−30.7 (6)
C4—N1—C2—C1	−179.9 (3)	N3—C8—C9—C14	−29.9 (6)
C4—N1—C3—S2	5.1 (6)	C7—C8—C9—C14	144.0 (4)
C2—N1—C3—S2	−177.5 (3)	C14—C9—C10—C11	−0.9 (6)
C4—N1—C3—S1	−177.0 (3)	C8—C9—C10—C11	174.0 (4)
C2—N1—C3—S1	0.4 (3)	C9—C10—C11—C12	0.6 (7)
C1—S1—C3—N1	−2.3 (3)	C10—C11—C12—C13	−0.1 (8)
C1—S1—C3—S2	175.8 (3)	C11—C12—C13—C14	−0.2 (8)
C3—N1—C4—O1	173.6 (4)	C12—C13—C14—C9	−0.1 (7)
C2—N1—C4—O1	−3.8 (5)	C10—C9—C14—C13	0.7 (6)
C3—N1—C4—C5	−4.9 (6)	C8—C9—C14—C13	−174.1 (4)
C2—N1—C4—C5	177.7 (4)	C8—N3—C15—C16	−54.4 (6)
C6—O2—C5—C4	77.9 (4)	N2—N3—C15—C16	110.5 (4)
O1—C4—C5—O2	1.1 (6)	C8—N3—C15—C20	128.9 (5)
N1—C4—C5—O2	179.6 (3)	N2—N3—C15—C20	−66.3 (5)
N3—N2—C6—O2	178.4 (4)	C20—C15—C16—C17	−0.9 (7)
N3—N2—C6—C7	0.9 (5)	N3—C15—C16—C17	−177.5 (4)
C5—O2—C6—N2	15.6 (6)	C15—C16—C17—C18	0.9 (7)
C5—O2—C6—C7	−167.1 (4)	C16—C17—C18—C19	−0.1 (7)
N2—C6—C7—C8	−0.6 (5)	C17—C18—C19—C20	−0.7 (7)
O2—C6—C7—C8	−178.1 (4)	C16—C15—C20—C19	0.1 (7)
N2—N3—C8—C7	0.5 (4)	N3—C15—C20—C19	176.7 (4)
C15—N3—C8—C7	166.1 (4)	C18—C19—C20—C15	0.7 (7)
N2—N3—C8—C9	175.6 (4)		
